# Supplementary material for: Switching from Intravenous to Subcutaneous Biological Therapy for Inflammatory Bowel Disease Patients Remains a Challenge
Source: J Clin Med. 2024 Feb 27;13(5):1357. doi: 10.3390/jcm13051357 (PMC10932065; doi:10.3390/jcm13051357)
Supplement: Supplementary file 1 [file jcm-13-01357-s001.zip › Questionnaire S1.pdf]

Supplementary S1. Questionnaire

Demographic and disease characteristics questionnaire

1. Gender: Male / Female
2. Year of Birth: __________
3. Weight: _____ Height: ____
4. Smoking: Yes / No
5. What is your religion:
    - Jewish
    - Muslim
    - Christian
    - Other
6. The type of inflammation I have: Ulcerative Colitis / Crohn's Disease
7. At what age was Crohn's disease / ulcerative colitis diagnosed? __________
8. Mark Employment Status:
    - Full-time Employee/Volunteer
    - Part-time Employee/Volunteer
    - I am not working now.
9. Which health insurance fund do you belong to?
    - Maccabi
    - Clalit
    - Mehuchedet
    - Leumit
10. Do you come to the hospital independently? Yes / No
11. Residence - Who do you live with?
    - Alone
    - With other people (spouse/family member/caregiver
12. Is your Crohn's disease/ulcerative colitis currently active? Yes / No
13. Are you currently being treated with steroids?
    - Yes, what time? ___________
    - No
14. Have you undergone surgeries related to Crohn's/Colitis?
    - Yes. How many surgeries?
    - No
15. Do you suffer from background diseases? Yes / No
    If yes, please select one of the following diseases?
    - Diabetes
    - Hypertension
    - Liver disease
    - Kidney disease
    - Heart failure
    - Ischemic heart disease
    - Asthma/COPD/other lung disease
    - Peripheral vascular disease
    - Stroke
    - Peptic ulcer disease (stomach/duodenum
    - Cancer

- HIV/AIDS
- Limb amputation
- Rheumatologic disease

Treatment preferences and experience questionnaire

1. What form is your current treatment?
    - Pills (Oral)
    - Subcutaneous injections (S.C)
    - Intravenous infusion (I.V)
    - I am not receiving treatment.
2. In what form have you been receiving treatment for your disease so far? Check all forms of treatment you have received:
    - Pills (Oral)
    - Subcutaneous injections (S.C)
    - Intravenous infusion (I.V)
3. Have you ever taken a certain medication in an irregular manner? (Delayed or not in the correct dosage)? Yes / No
4. If yes, how was the medication administered?
    - Pills (Oral)
    - Subcutaneous injections (S.C)
    - Intravenous infusion (I.V)
5. If it were possible to administer any medication in any form without a difference in the medication's cost, which form of administration would you prefer?
    - Rate the preference for treatment with pills (Oral) once a day:
      Least preferred 1 ☐ 2 ☐ 3☐ 4 ☐ Most preferred

    - Rate the preference for subcutaneous injections (S.C) every 2 weeks:
      Least preferred 1 ☐ 2 ☐ 3☐ 4 ☐ Most preferred

    - Rate the preference for subcutaneous injections (S.C) every 8 weeks:
      Least preferred 1 ☐ 2 ☐ 3☐ 4 ☐ Most preferred

    - Rate the preference for intravenous infusion (I.V) every 8 weeks:
      Least preferred 1 ☐ 2 ☐ 3☐ 4 ☐ Most preferred

6. If you are currently receiving intravenous infusion, would you be interested in receiving the same medication through subcutaneous injection? Yes / No
7. If you prefer intravenous infusion over subcutaneous injection, state the reasons for preferring infusion (Multiple answers are possible):
    - Unable to self-inject/fear of needles
    - Prefer medical staff to be present during treatment
    - Want to meet a doctor after each treatment
    - Connection with other patients, social/information exchange during infusion
    - Believe that infusion is more effective
    - Longer interval between infusions
8. Based on your experience, how much time does the entire process of receiving infusion take on average, including travel time, waiting, the infusion itself, post-infusion waiting, and travel time back home/work?

- Did not receive infusion
- Up to 3 hours
- Up to 4 hours
- Up to 5 hours
- Up to 6 hours
- More than 6 hours

9. If you prefer subcutaneous injection over intravenous infusion, state the reasons for preferring infusion (Multiple answers are possible):
    - Not preferred
    - Easier self-administration at home
    - Difficulty finding a vein for infusion
    - Difficulty getting to the hospital
    - Don't want to lose a workday
    - Time-saving
    - Want to avoid exposure to infections by not going to the hospital
    - No availability of appointments for infusion
10. To what extent does the cost of the medication influence your decision in choosing the administration form?
    - Very much
    - A lot
    - To some extent
    - Little
    - Not at all
