# Supplementary material for: Switching from Intravenous to Subcutaneous Biological Therapy for Inflammatory Bowel Disease Patients Remains a Challenge
Source: J Clin Med. 2024 Feb 27;13(5):1357. doi: 10.3390/jcm13051357 (PMC10932065; doi:10.3390/jcm13051357)
Supplement: Supplementary file 1 [file jcm-13-01357-s001.zip › Table S1.pdf]

Table S2. Patients' Preferences: A Comparison between IV Treatment Every 8 Weeks and SC Treatment Every Two Weeks.

Characteristics	Patients prefer IV every 8w n=157	Patients prefer SC every 2w n =187	No particular preference n=110	*p*-value
Age (median, (IQR))	36 (26-52) [a]	46 (31-59) [b]	44 (33.5-59) [b]	0.001
Female (n, %)	83 (52.9)	104 (55.6)	66 (60)	0.515
BMI (median, (IQR))	24.1 (21.3-26.8) [b]	25.1 (21.7-29.1) [a]	23.9 (21.1-26.6) [b]	0.037
Independent arrival to day care (n, %)	145 (92.4)	165 (88.2)	104 (94.5)	0.157
Dwelling with other people	138 (87.9)	158 (84.5)	101 (91.8%)	0.189
Infusion Process Time (Mean, (SD))	4.37 (1.12)	4.63 (1.29)	4.57 (1.29)	0.278
Employment status				
Currently not employed (n, %)	45 (28.7)	60 (32.1)	33 (30.0)	0.918
Full-time job (n, %)	88 (56.1)	95 (50.8)	59 (53.6)	
Part-time job (n, %)	24 (15.3)	32 (17.1)	18 (16.4)	
Another systemic disease* (n, %)	52 (33.1)	68 (36.4)	45 (40.9)	0.426
IBD type				
Crohn's disease (n, %)	96 (61.1) [a]	140 (74.9) [b]	71 (64.5) [ab]	0.017
Ulcerative colitis (n, %)	61 (38.8) [a]	47 (25.1) [b]	39 (35.5) [ab]	
Age of diagnosis (median, (IQR))	24 (17-34) [a]	26 (19-41) [b]	25 (20-40.2) [b]	0.030
Active disease (n, %)	69 (43.9)	97 (51.9)	57 (51.8)	0.274
Quality of Life score (median, (IQR))	21 (11-35.5)	25 (15-35)	25 (11.7-34.5)	0.474

Abbreviations: IBD, inflammatory bowel disease; IQR, interquartile range; SD, standard deviation; w, weeks.

[1]Another systemic disease included: Lung, liver, kidney, ischemic heart disease, congestive heart disease, peripheral vascular disease, diabetes mellites, cerebrovascular disease, rheumatic diseases, peptic ulcer disease.

[2] a, b Each subscript letter denotes a subset category whose column proportions do not differ significantly from each other at the p=0.05 level.
