# Supplementary material for: Switching from Intravenous to Subcutaneous Biological Therapy for Inflammatory Bowel Disease Patients Remains a Challenge
Source: J Clin Med. 2024 Feb 27;13(5):1357. doi: 10.3390/jcm13051357 (PMC10932065; doi:10.3390/jcm13051357)
Supplement: Supplementary file 1 [file jcm-13-01357-s001.zip › Table S2.pdf]

Table S2. Cost comparison table of maintenance treatment.

Drug	Comparator	Recommended dosage	Price ($)	Additional payments per treatment ($)	Annually Total Cost ($)
Vedolizumab (Entyvio)	Vial for IV infusion	300 mg every 8 weeks	1959	Infusion center - 110<br>Loss of workday-162<br>Public transport/ Parking – 5.50<br>Total – 277.5 (Annualy-1665)	13,419
	Pen for SC injection	108 mg every 2 weeks	957	None	22,968
Infliximab biosimilar (Remsima)	Vial for IV infusion	5 mg/kg every 8 weeks – On average 400 mg every 8 weeks	1968	Infusion center - 110<br>Loss of workday-162<br>Public transport/ Parking – 5.50<br>Total – 277.5 (Annualy-1665)	13,473
	Pen for SC injection	120 mg every 2 weeks	272	None	6,528
